# Premotor projections from the locus coeruleus and periaqueductal grey are altered in two rat models with inborn differences in emotional behavior

**DOI:** 10.1007/s00221-024-06786-y

**Published:** 2024-02-15

**Authors:** Elizabeth A. Shupe, Ilan A. Kerman, Sarah M. Clinton

**Affiliations:** 1 School of Neuroscience, Virginia Tech, Blacksburg, VA 24061 USA; 2 Behavioral Service Line, Veterans Affairs Minneapolis Health Care, Minneapolis, MN USA

**Keywords:** Periaqueductal grey, Locus coeruleus, Somatomotor, Pseudorabies, Rat

## Abstract

Emotionally motivated behaviors rely on the coordinated activity of descending neural circuits involved in motor and autonomic functions. Using a pseudorabies (PRV) tract-tracing approach in typically behaving rats, our group previously identified descending premotor, presympathetic, and dual-labeled premotor-presympathetic populations throughout the central rostral-caudal axis. The premotor-presympathetic populations are thought to integrate somatomotor and sympathetic activity. To determine whether these circuits are dysregulated in subjects with altered emotional regulation, subsequent neuroanatomical analyses were performed in male subjects of two distinct genetic models relevant to clinical depression and anxiety: the Wistar Kyoto (WKY) rat and selectively bred Low Novelty Responder (bLR) rat. The present study explored alterations in premotor efferents from locus coeruleus (LC) and subdivisions of the periaqueductal grey (PAG), two areas involved in emotionally motivated behaviors. Compared to Sprague Dawley rats, WKY rats had significantly fewer premotor projections to hindlimb skeletal muscle from the LC and from the dorsomedial (DMPAG), lateral (LPAG), and ventrolateral (VLPAG) subdivisions of PAG. Relative to selectively bred High Novelty Responder (bHR) rats, bLR rats had significantly fewer premotor efferents from LC and dorsolateral PAG (DLPAG). Cumulatively, these results demonstrate that somatomotor circuitry in several brain areas involved in responses to stress and emotional stimuli are altered in rat models with depression-relevant phenotypes. These somatomotor circuit differences could be implicated in motor-related impairments in clinically depressed patients.

## Introduction

Numerous behaviors in humans and animals depend upon intricate coordination of the somatic motor and autonomic nervous systems. Although plenty of these behaviors occur in the absence of stress, this synchronized outflow of motor and autonomic efferents is often engaged as part of a broader response to changes in an organism’s environment. A cornerstone example of this phenomenon is the classic fight-or-flight response, an emotionally motivated stress response in which an organism encounters a predator or rival and must prepare to fight or flee to ensure survival. The defensive-aggressive fight-or-flight response is distinguished by active stress coping strategies involving a combination of somatomotor actions (e.g., alerting, hissing, and sudden attack behavior) and concomitant autonomic changes (e.g., elevated blood pressure and cardiac output) (Mancia and Zanchetti 1981; Hilton 1982; Jordan 1990; Kerman et al. 2006). The contrasting actions involved in a passive stress coping approach are characterized by their own distinct behavioral and physiological features, including immobilization, enhanced corticosteroid secretion, and changes in blood pressure (Mancia and Zanchetti 1981; Kerman et al. 2006). Together, these examples and others underscore the necessity for organisms to have a means of integrating the parallel activities of the somatic motor and autonomic nervous systems (Kerman 2008).

Mechanisms governing central control of motor and autonomic outflows were traditionally considered to be quite distinct from one another (Kerman 2008). Actions of the somatic motor system, which innervates and commands skeletal muscle fibers, were thought to be largely voluntary (Kandel et al. 2000). By contrast, autonomic functions are thus named because they occur automatically and without conscious control (Langley 1921; Cannon 1963). This assumption has since been challenged in anatomical studies by Holstege and colleagues, who were the first to describe an emotional motor system responsible for coordinating somatomotor activity during emotionally motivated behaviors (Holstege and Kuypers 1987; Holstege 1995, 1996). Although this system is organized similarly to the somatic motor system, structures comprising the emotional motor system also receive afferents from several integrative limbic structures and contain neurons that regulate autonomic functions (Loewy 1990; Aston-Jones et al. 1995; Holstege 1995; Kerman 2008).

In earlier work, our laboratory group investigated whether integration of somatomotor-sympathetic efferents was achieved by populations of neurons with singular functional roles in common neuroanatomical nodes of this circuit or by populations of neurons with dual functions. Using typically behaving male Sprague Dawley rats, we adopted a retrograde viral tract-tracing strategy to separately label transsynaptic connections from peripheral motor and sympathetic targets (Kerman et al. 2003, 2006; Kerman 2008). Two genetically modified strains of pseudorabies virus (PRV), PRV-152 and PRV-BaBlu, were injected into our selected motor target (sympathectomized hindlimb gastrocnemius muscle) and our sympathetically innervated target (adrenal gland), respectively. These experiments revealed the presence of discrete somatomotor and sympathetic neurons, as well as putative dual-function somatomotor-sympathetic neurons (SMSNs) infected by both recombinant PRV strains, throughout the central rostral-caudal axis (Kerman 2008). The majority of caudal SMSNs were concentrated in subareas of the ventral medulla, although a small number of caudal SMSNs were also located in locus coeruleus (LC) and the A5 and A7 noradrenergic cell groups (Kerman et al. 2003). Rostrally located SMSNs were observed in several regions, with a majority concentrated in subdivisions of the periaqueductal gray (PAG; caudal and intermediate ventrolateral portions) and hypothalamus (dorsomedial (DMH), dorsolateral lateral (dlLH), and medial parvocellular ventral subdivision of the paraventricular nucleus (PVNmpv)) (Kerman et al. 2006). In agreement with prior work using classical tract-tracing methods, these studies found that ventromedial medulla, ventrolateral PAG (VLPAG), PVN, and LH contained direct projections to spinal cord. Higher-order SMSNs were distributed in several additional areas, including DMH (which has projections to PVN, VLPAG, and ventromedial medulla) and other subdivisions of the PAG (Kerman et al. 2003, 2006; Kerman 2008).

A notable finding that emerged from these experiments was that several areas involved in somatomotor-sympathetic integration were key structures involved in the regulation of organismal responses to stressful stimuli. Perturbations of the stress response are commonly observed in patients with mood and anxiety disorders (Nestler et al. 2002; Tafet and Nemeroff 2020). Recent work by our laboratory investigated whether somatomotor-sympathetic circuitry of the PVN, an key integrator of behavioral, endocrine, and autonomic responses to stress (Herman and Cullinan 1997), is altered in rodent models with emotional behavior dysregulation (Shupe et al. 2021). Our PRV-mediated neuroanatomical investigations in male Wistar-Kyoto (WKY) and selectively bred Low Novelty Responder (bLR) rats, two models featuring inborn differences in emotionality and stress reactivity (Stead et al. 2006; Stedenfeld et al. 2011; Nam et al. 2014; Burke et al. 2016; Prater et al. 2017), revealed that both strains display fewer descending somatomotor projections from the PVN, potentially contributing to the locomotor disturbances associated with their altered emotional states (Shupe et al. 2021). In the present study, we continued our investigations in the WKY and bLR models to explore the extent to which premotor midbrain and brainstem circuitry differ in these models relative to their respective controls. Using PRV transsynaptic tract-tracing, the following experiments evaluated descending somatomotor circuits in two additional structures relevant to stress and emotional regulation: the PAG and LC. Based on our previous observations in PVN, we hypothesized that fewer premotor projections to hindlimb skeletal muscle would be observed in subdivisions of the PAG and LC of WKY and bLR rats.

## Methods

Experiments described in this report were approved by the Institutional Committee on the Use and Care of Animals (IACUC) and performed in accordance with the National Institutes of Health guidelines on animal research (USA, 2011).

### Animals

Adult male WKY and Sprague Dawley rats were purchased from Charles River Laboratories (Kingston, NY, USA). Adult male selectively bred Low and High Novelty Responder rats were obtained from the fifteenth generation of the in-house breeding colony. The process of generating and characterizing the selectively bred HR and LR lines has been described elsewhere (Stead et al. 2006; Flagel et al. 2014). Validation of the emotional behavior phenotypes in these strains was performed previously (Shupe et al. 2021). Housing was maintained under conditions of standard temperature (21–23 °C) and humidity (50–55%) in a 12:12 h light–dark cycle (lights on at 6:00 a.m.). Rats were housed in groups of 2–3 animals per cage and provided ad libitum chow and water.

## Experiment 1: Viral tract-tracing of PAG and LC premotor efferents in WKY rats versus outbred Sprague Dawley rats

### Viral tract-tracing overview

Consistent with our group’s prior work, the first experiment employed retrograde viral tract-tracing to label premotor and presympathetic efferents in a cohort of WKY and Sprague Dawley rats (*n* = 9 per strain) as performed previously (Shupe et al. 2021). To this end, two transgenic recombinants of an attenuated PRV strain, PRV-Bartha, were used: (1) PRV-152, which contains the gene encoding enhanced green fluorescent protein (eGFP), and (2) PRV-BaBlu, which carries the lac Z gene at the gG locus and codes for β-galactosidase (β-gal) (Kim et al. 1999; Smith et al. 2000). In the present study, PRV-152 was injected into sympathectomized gastrocnemius muscle (i.e., skeletal muscle; selected motor target) while PRV-BaBlu was injected into the adrenal gland (i.e., selected sympathetic target) as described in earlier work by our laboratory (Kerman et al. 2006, 2007; Kerman 2008; Shupe et al. 2021). Due to technical challenges related to the β-gal primary antibody as well as practical consideration of the lack of differences in immunolabeling of presympathetic connections previously recorded in the PVN (Shupe et al. 2021), analyses in the present study were limited to immunolabeling of eGFP-expressing premotor-connected cells.

### Sympathectomy and viral injections

Procedures were performed as described previously (Kerman et al. 2003, 2006; Shupe et al. 2021). Rats were anesthetized with 5% isoflurane vaporized in 1.0–1.5 L/min of O_2_ maintained at 1.5–2.5%. Verification of surgical plane of anesthesia was determined by absence of spontaneous movement and lack of withdrawal responses to tail and/or foot pinch. Surgical removal of sympathetic innervation to the hindlimb musculature was performed prior to PRV injections as previously described (Kerman et al. 2003, 2006; Shupe et al. 2021). In brief, the lumbar sympathetic nerve was dissected by ventral laparotomy and a segment of the lumbar sympathetic nerve from the renal artery to the aortic bifurcation was extirpated. Neural plexuses along the descending aorta were removed with fine forceps under microscopic observation, and the aorta was swabbed with a 10% phenol solution. This procedure is effective at removing a large majority of sympathetic hindlimb efferents and has been performed successfully numerous times (Kerman et al. 2003, 2006, 2007; Shah et al. 2013; Shupe et al. 2021).

Viral recombinants used in the present study were harvested from pig kidney cell cultures at a titer of 10^8^–10^9^ pfu/mL and aliquoted into 50-μL volumes. Viral stocks were stored at -80 °C until the time of inoculation, at which time they were rapidly thawed in a 37 °C water bath. Following surgical sympathectomy and a 2–10-day recovery period, rats were anesthetized and injected with PRV-152 and PRV-BaBlu as described previously (Kerman et al. 2003, 2006, 2007; Shah et al. 2013; Shupe et al. 2021). In brief, 1-μL volumes of PRV-152 totaling 30 μL were injected throughout the lateral head of the left gastrocnemius muscle using a 10-μL glass syringe (Hamilton Company, Reno, NV, USA). After a period of 24 h, 2–4 μL of PRV-BaBlu was similarly injected into the ipsilateral adrenal gland using a Hamilton syringe with a glass pipette attached to the tip with wax. Our laboratory determined previously that 12–24 h of separation between gastrocnemius and adrenal gland injections is necessary to match the temporal transport of the two viral recombinants (Kerman et al. 2003, 2006). Analgesic buprenorphine (0.05–0.1 mg/kg, s.c.) was administered during all surgical procedures. Carpofen (5 mg/kg, s.c.) was administered once daily for post-operative pain relief.

### Tissue processing

At 132 h following the initial PRV injections, tissue collection and processing procedures were performed as described previously (Shupe et al. 2021). Rats were deeply anesthetized with sodium pentobarbital (150 mg/kg) and transcardially perfused. Brains and spinal cords were extracted, post-fixed overnight, and immersed in 30% sucrose overnight the following day. Brains were sectioned coronally at a thickness of 40 μm on a freezing microtome and stored in cryoprotectant (30% ethylene glycol, 1% polyvinyl-pyrrolidone, 30% sucrose in 0.1 M sodium phosphate buffer (Kerman et al. 2003)) until immunohistochemical processing. Spinal cords were extracted in three segments (T1–T7, T8–T13, and L1–L6), and postfixed and processed as above for brain samples. Spinal cord segments were sectioned horizontally at a thickness of 40 μm and stored at  – 20 °C in cryoprotectant.

In the present study, a subset of brains of PRV-injected rats were processed for immunofluorescent detection of eGFP (*n* = 5 per strain). Free-floating coronal brain sections spanning Bregma levels  – 6.72 mm to  – 14.76 mm were washed several times at room temperature with 1X phosphate buffered saline (PBS; pH 7.4) followed by incubation for one hour in blocking solution (0.2% Triton X-100 and 2% gelatin from cold water fish skin in 1X PBS). Sections were then incubated in primary antibody solution (chicken anti-GFP (Cat. No. ab13970, Abcam, Cambridge, MA, USA), 1:2,000 in blocking solution, for one hour at room temperature before incubating overnight at 4 °C. The next day, tissue was rinsed several times in 1X PBS at room temperature before incubating in secondary antibody solution for two hours (AlexaFluor 488 goat anti-chicken IgY H + L (Cat. No. A11039, Invitrogen/Life Technologies Corporation, Eugene, OR, USA), 1:200, in blocking solution). Following additional washes in 1X PBS, tissue was mounted to glass slides and coverslipped with VectaShield Antifade Mounting Medium with DAPI (Cat. No. H-1200, Vector Laboratories, Newark, CA, USA) and stored at 4 °C.

### Tissue analysis

Whole brain sections were imaged using a Keyence BZ-X810 All-in-One Fluorescence Microscope equipped with a computer-controlled motorized stage (Keyence Corporation of America, Itasca, IL, USA). Images were digitized under a 20 × objective with specific filter sets corresponding to DAPI (excitation and emission ranges 325–375 nm and 435–485 nm, respectively; Cat. No. 49000-UF1, Chroma Technology Corporation, Bellows Falls, VT, USA) and eGFP (excitation and emission ranges 450–490 nm and 500–550 nm, respectively; Cat. No. 49002-UF1, Chroma Technology Corporation). Images were acquired in multiple z-stacks and stitched together under Full Focus in BZ-X Analyzer software (Keyence).

The Rat Brain in Stereotaxic Coordinates was used as a reference for anatomical classification of individual regions of interest (Paxinos and Watson 2007). Bilateral quantification of cells expressing eGFP and measurements of area (mm^2^) was performed manually in FIJI software (Schindelin et al. 2012) for consecutive sections sampled at 240-µm intervals. Where necessary, co-expression of DAPI was used to confirm the location of discrete cell somas. eGFP-expressing cells were quantified in the LC (Bregma levels  – 9.48 mm to  – 10.32 mm) and PAG (Bregma levels  – 6.72 to  – 8.64). Discrete quantifications of eGFP-expressing cells were performed for four subdivisions of the PAG: (1) dorsomedial (DMPAG; Bregma levels  – 6.72 mm to  – 8.76 mm); (2) dorsolateral (DLPAG; Bregma levels  – 6.72 mm to  – 8.16 mm); (3) lateral (LPAG; Bregma levels  – 6.72 mm to  – 8.64 mm); and (4) ventrolateral (VLPAG; Bregma levels  – 6.72 mm to  – 8.76 mm). Analyses in the PAG focused on the caudal two-thirds of this region, as this portion of the PAG is implicated in active and passive stress coping strategies (Keay and Bandler 2015). Of the subset of samples used for immunofluorescence processing (*n* = 5 per group), one WKY sample was excluded from analysis due to poor tissue quality after processing.

Figures were prepared using BZ-X Analyzer software and Adobe Illustrator 2023 (Adobe Inc., San Jose, CA).

## Experiment 2: Viral tract-tracing of PAG and LC premotor efferents in bLR versus bHR rats

In complement to Experiment 1, a separate cohort of bHR and bLR rats (*n* = 7 per group) were used in a parallel PRV tract-tracing study. PRV injections were performed as previously described for Experiment 1. Rats were allowed to survive 120 h post-injection. Subsequent procedures for euthanasia, tissue collection, and tissue processing were performed in a manner identical to those described for Experiment 1. eGFP immunofluorescence was carried out in a subset of tissue samples (*n* = 5 per group). From this subset of samples, two samples (*n* = 1 per group) were excluded from final analysis due to an absence of PRV immunolabeling throughout the rostral-caudal axis.

### Statistical analysis

**Fig. 1:**
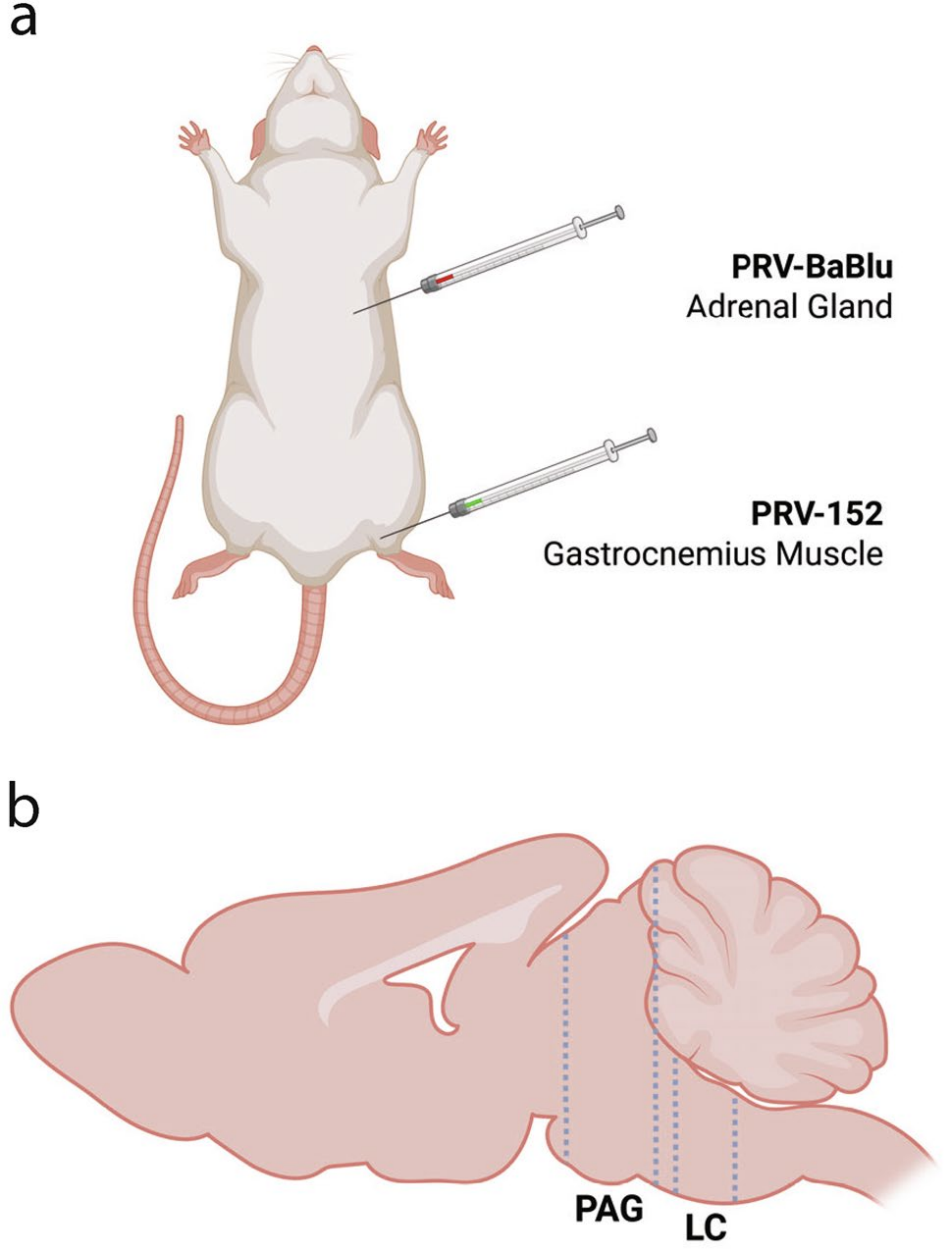
Schematic of pseudorabies virus (PRV) injection protocol and brain areas examined in the present study. **a** In adult male rats, PRV-152 was injected into sympathectomized gastrocnemius muscle (i.e., skeletal muscle; selected motor target). PRV-BaBlu was injected into the adrenal gland (i.e., selected sympathetic target) 24 h later. Animals were sacrificed after 120 (bHR/bLR) or 132 h (SD/WKY) and their brains were harvested for subsequent analysis. **b** Immunofluorescent detection of premotor PRV-infected cells was assessed in the caudal two-thirds of the periaqueductal grey (PAG) and in the locus coeruleus (LC). Created with BioRender.com

GraphPad Prism Version 9.5.1 (GraphPad Software) was used for statistical analysis. For each animal, eGFP cells per mm^2^ for each region of interest was calculated as the sum of total bilateral cell counts from all brain sections divided by the total area (mm^2^) over which counts were performed to account for disparities in the number of tissue sections surveyed between samples. Data were assessed for normality prior to analysis using the Shapiro–Wilk test. Data were excluded only if determined to be an outlier using both the Grubbs test and the ROUT method. When data were normally distributed, differences between WKY versus SD rats and bLR versus bHR rats were analyzed using two-tailed Welch’s t-tests. The Mann–Whitney U test (two-tailed) was used when data were not normally distributed. All results are expressed as mean ± SEM. Significance was set at *p* < 0.05 (Fig. 1).

## Results

### Experiment 1

#### Quantitative analyses of PRV-152 immunolabeling in WKY versus Sprague Dawley rats

**Fig. 2:**
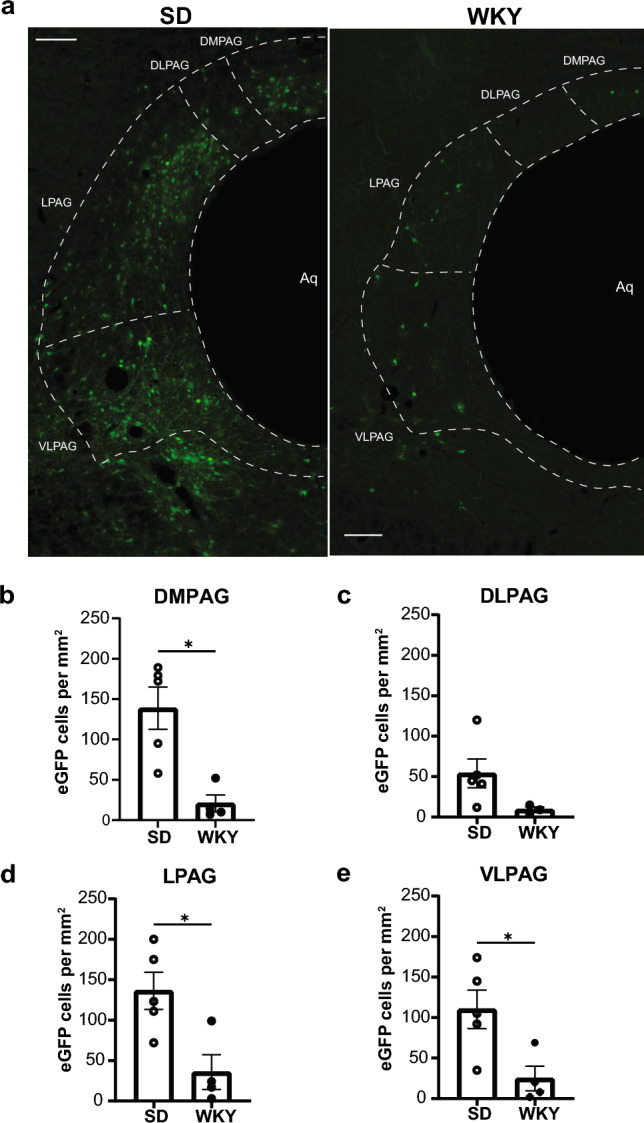
Pseudorabies virus (PRV) immunolabeling of premotor (PRV-152) cells in the periaqueductal gray (PAG) of adult male Sprague Dawley (SD) and Wistar-Kyoto (WKY) rats. **a** Representative images of transsynaptically labeled cells infected with PRV-152 in subdivisions of the PAG in SD rats ( – 8.04 mm Bregma) and WKY rats ( – 7.80 mm Bregma) that survived 132 h after injections with PRV-152 and PRV-BaBlu. The aqueduct (Aq) is oriented to the right side of each image. Scale bar, 200 µm. **b** Quantification of somatomotor projecting cells in dorsomedial (DM)PAG. **c** Quantification of somatomotor projecting cells in dorsolateral (DL)PAG. **d** Quantification of somatomotor projecting cells in lateral (L)PAG. **e** Quantification of somatomotor projecting cells in ventrolateral (VL)PAG. Bars represent mean ± SEM. Statistically significant differences at *p < 0.05

We first examined eGFP immunolabeling of cells with premotor projections to gastrocnemius muscle in the dorsomedial (DMPAG), dorsolateral (DLPAG), lateral (LPAG), and ventrolateral (VLPAG) subdivisions of the PAG in WKY and Sprague Dawley rats (Fig. 2). Relative to Sprague Dawley rats, WKY rats showed significant reductions in the quantity of premotor projecting cells per unit area in the DMPAG (Mann–Whitney test, *p* = 0.0159; Fig. 2b), LPAG (*t*(6.954) = 3.194, *p* = 0.0153; Fig. 2d), and VLPAG (*t*(6.496) = 3.028, *p* = 0.0210; Fig. 2e). Although a smaller quantity of premotor efferent cells per unit area were also observed in the DLPAG of WKY rats relative to Sprague Dawley rats, the data did not meet the threshold for significance (*t*(4.249) = 2.463, *p* = 0.0658; Fig. 2c). Density of premotor immunolabeling was greatest at Bregma levels  – 7.56 mm to  – 8.76 mm in the DMPAG; between  – 7.68 mm to  – 8.16 mm in the DLPAG; between  – 7.32 mm to  – 7.92 mm in the LPAG; and between  – 7.20 mm to  – 8.04 mm in the VLPAG. Overall immunolabeling of premotor projections in the DLPAG was sparse for both strains.

**Fig. 3:**
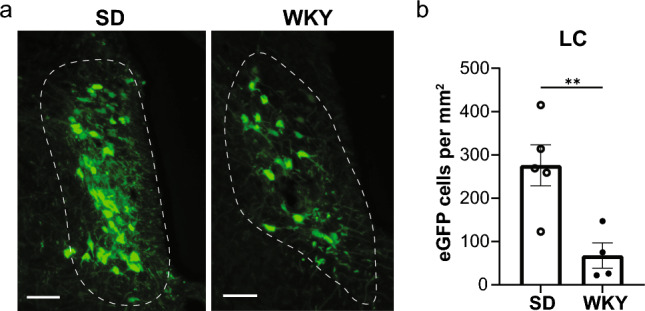
Pseudorabies virus (PRV) immunolabeling of premotor (PRV-152) cells in the locus coeruleus (LC) of adult male Sprague Dawley (SD) and Wistar-Kyoto (WKY) rats. **a** Representative images of transsynaptically labeled cells infected with PRV-152 in the LC of SD rats ( – 9.96 mm Bregma) and WKY rats ( – 9.72 mm Bregma) that survived 132 h after injections with PRV-152 and PRV-BaBlu. The fourth ventricle (4 V) is oriented to the right side of each image. Scale bar, 100 µm. **b** Quantification of somatomotor projecting cells in LC. Bars represent mean ± SEM. Statistically significant differences at *p < 0.05

Next, we quantified immunolabeling of muscle-connected cells in the LC (Fig. 3). Mirroring the findings in PAG, the LC contained fewer premotor efferent cells in WKY rats compared to Sprague Dawley rats (*t*(6.391) = 3.761, *p* = 0.0084; Fig. 3b). Greater density of immunolabeling of cells infected with PRV-152 in both strains was predominantly observed in the rostral LC (corresponding to Bregma levels  – 9.48 mm to  – 9.84 mm).

### Experiment 2

#### Quantitative analyses of PRV-152 immunolabeling in bLR versus bHR rats

**Fig. 4:**
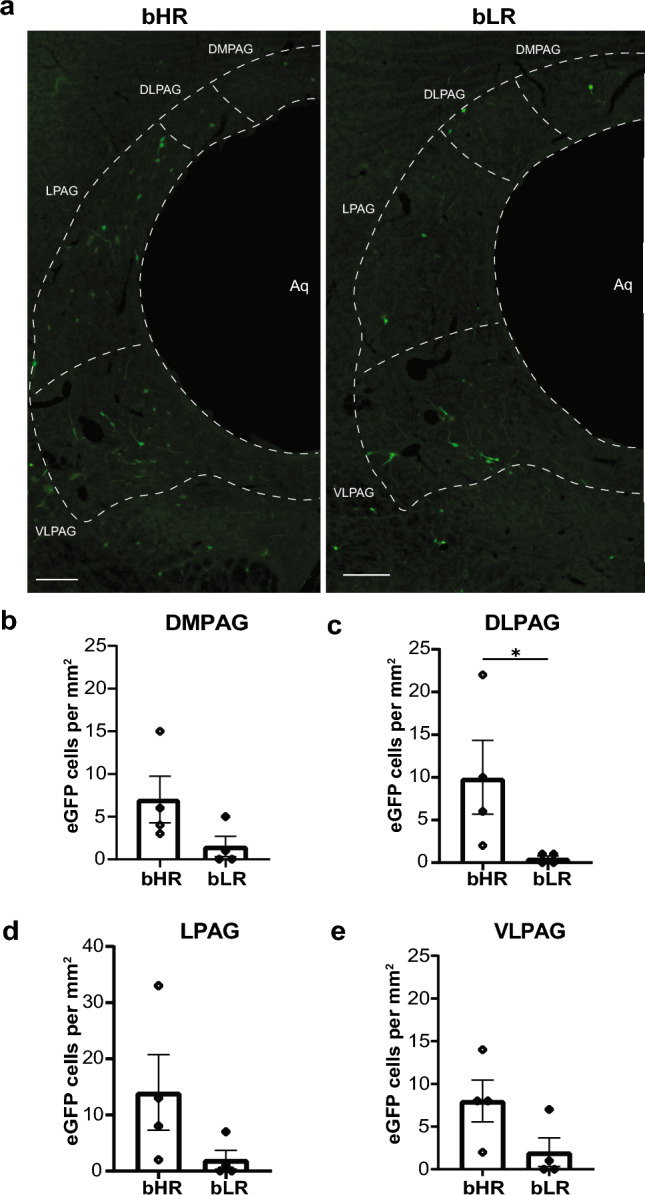
Pseudorabies virus (PRV) immunolabeling of premotor (PRV-152) cells in the periaqueductal gray (PAG) of adult male selectively bred High Novelty Responder (bHR) and Low Novelty Responder (bLR) rats. **a** Representative images of transsynaptically labeled cells infected with PRV-152 in subregions of the PAG in bHR rats ( – 8.04 mm Bregma) and bLR rats ( – 8.04 mm Bregma) that survived 120 h after injections with PRV-152 and PRV-BaBlu. The aqueduct (Aq) is oriented to the right side of each image. Scale bar, 200 µm. **b** Quantification of somatomotor projecting cells in dorsomedial (DM)PAG. **c** Quantification of somatomotor projecting cells in dorsolateral (DL)PAG. **d** Quantification of somatomotor projecting cells in lateral (L)PAG. **e** Quantification of somatomotor projecting cells in ventrolateral (VL)PAG. Bars represent mean ± SEM. Statistically significant differences at *p < 0.05

In complement to the assessments performed in Experiment 1, immunolabeling of premotor efferents was first quantified in subdivisions of the PAG in bLR rats relative to bHR rats (Fig. 4). Significantly fewer premotor projections to gastrocnemius muscle per unit area were observed in the DLPAG of bLR rats compared to bHR rats (Mann–Whitney test, *p* = 0.0286; Fig. 4c). Trends for a significant decrease in immunolabeling of premotor efferents per unit area were observed in LPAG (Mann–Whitney test, *p* = 0.0571; Fig. 4d) and VLPAG (Mann–Whitney test, *p* = 0.0571; Fig. 4e). Significant differences in immunolabeling of premotor efferents per unit area were not observed in the DMPAG (*t*(4.094) = 1.842, *p* = 0.1376; Fig. 4b). Density of premotor immunolabeling was most prominently observed between Bregma levels  – 7.32 mm to  – 7.56 mm in the DMPAG; at  – 7.20 mm in the DLPAG; between  – 7.44 mm to  – 8.04 mm in the LPAG; and between  – 7.44 mm to  – 7.56 mm and  – 7.92 mm to  – 8.16 mm in the VLPAG.

**Fig. 5:**
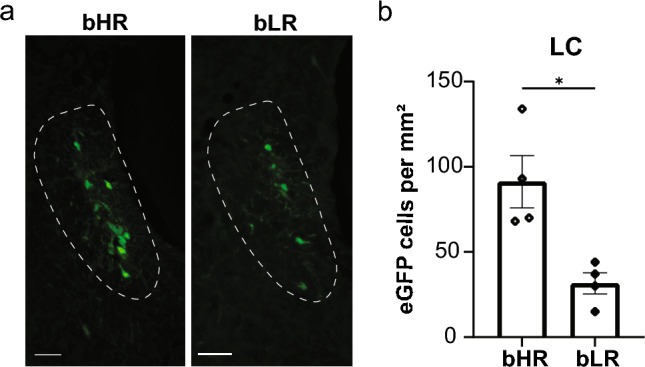
Pseudorabies virus (PRV) immunolabeling of premotor (PRV-152) cells in the locus coeruleus (LC) of adult male selectively bred High Novelty Responder (bHR) and Low Novelty Responder (bLR) rats. **a** Representative images of transsynaptically labeled cells infected with PRV-152 in the LC of bHR rats ( – 9.84 mm Bregma) and bLR rats ( – 9.84 mm Bregma) that survived 120 h after injections with PRV-152 and PRV-BaBlu. The fourth ventricle (4 V) is oriented to the right side of each image. Scale bar, 100 µm. **b** Quantification of somatomotor projecting cells in LC. Bars represent mean ± SEM. Statistically significant differences at *p < 0.05

Finally, immunolabeling of premotor projections in LC was examined for bHR and bLR rats (Fig. 5). Relative to bHR rats, bLR rats had significantly fewer PRV-152-infected cells per unit area in the LC (*t*(3.954) = 3.612, *p* = 0.0230; Fig. 5b). As in WKY and Sprague Dawley rats, greater densities of eGFP-expressing cells in the LC of bHR and bLR rats were primarily observed in the rostral LC (corresponding to Bregma levels -9.48 mm to -9.84 mm).

## Discussion

The present study extended our neuroanatomical investigations of polysynaptic somatomotor projections from central structures to skeletal muscle in two rat models that innately display behavioral and physiological traits relevant to emotional disorders. Using a PRV tract-tracing approach, we demonstrated that male WKY and bLR rats both have fewer descending somatomotor projections from LC and various subdivisions of the PAG. Specifically, male WKY rats have significantly fewer muscle-projecting neurons from the LC, DMPAG, LPAG, and VLPAG (relative to outbred Sprague Dawley rats), and male bLR rats have significantly fewer muscle-projecting neurons from the LC and DLPAG (compared to bHR rats). Despite these models being derived from distinct genetic backgrounds, these findings indicate that both strains share additional premotor circuit abnormalities beyond that documented previously by our group (Shupe et al. 2021). The viral tract-tracing data in PAG also suggest the possibility that some nodes of the somatomotor-sympathetic circuitry could be differentially regulated between these two model systems, which might contribute to observed variations between these strains.

Prior retrograde tract-tracing experiments by our laboratory demonstrated that the LC contains a small but notable quantity of neurons with dual premotor and presympathetic outflows (in addition to single-function neurons with these roles) in typically behaving male rats (Kerman et al. 2003). The LC modulates sympathetic arousal, cognitive and behavioral responses to stress, and other functions via noradrenergic signaling throughout the forebrain, brainstem, and spinal cord (Bangasser and Valentino 2014; Poe et al. 2020). LC neurons can shift their mode of activity between tonic and phasic firing, allowing an organism to shift attentional strategies (i.e., survey its surroundings or sustain focused attention) to adapt to changes in its environment (Aston-Jones and Cohen 2005; Bangasser et al. 2016). When the hypothalamic-pituitary-adrenocortical (HPA) axis is recruited during stress, corticotropin-releasing hormone (CRH) release by the PVN engages LC neurons to favor increased tonic firing (and decreased phasic firing) to promote greater sympathetic arousal and cognitive flexibility (Poe et al. 2020). Although investigations of the LC-norepinephrine (NE) system in the bLR model are not well-documented, some data are available in the WKY model. Electrophysiological investigations in WKY rats show that basal and phasic firing activity are elevated in these animals (Bruzos-Cidón et al. 2014). Additionally, WKY rats show attenuated NE release during acute stress as well as molecular signatures consistent with greater NE turnover (Pardon et al. 2002; Pearson et al. 2006). Indeed, forced swim test (FST) passive coping responses in the WKY rat are thought to be mediated largely by noradrenergic neurotransmission (Tejani-Butt et al. 1994; López-Rubalcava and Lucki 2000; Rittenhouse et al. 2002). By contrast, FST passive coping behavior in bLR rats is improved by modulation of serotonergic and noradrenergic signaling (Jama et al. 2008). The role of the LC in promoting a passive stress coping strategy in the WKY and bLR models could be related to observed deficits in stress-induced noradrenergic reactivity (Pearson et al. 2006; Bruzos-Cidón et al. 2014), independent of premotor efferent projections. However, the recent observations that male WKY and bLR rats have fewer premotor projections in both PVN and LC could also potentially contribute to a more passive behavioral approach (i.e., reduced locomotor activity) in response to stressful or novel situations by way of insufficient functional connectivity of premotor efferents to engage an adequate locomotor response.

In comparison to the LC, the PAG appears to be a more prominent node of the premotor-presympathetic circuitry (Kerman et al. 2003; Kerman 2008). The PAG is implicated in autonomic and behavioral responses to panic and threat as well as modulation and perception of pain (Johnson et al. 2008). This structure consists of dorsal and ventrolateral columns that span the rostral-caudal extent and mediate distinct stress coping strategies (Keay and Bandler 2015). Early functional studies in the PAG demonstrated that the caudal two-thirds of the dorsal PAG are involved in active defensive reactions, which are characterized by escape behaviors and elevated blood pressure, among other features. Whereas active coping strategies mediated by the DLPAG appear to be engaged by escapable psychological stressors, the activities of the LPAG are likely engaged by escapable physical stressors (Keay and Bandler 2015). By contrast, the ventrolateral portion of the caudal PAG promotes manifestations of passive stress coping (i.e., immobility, hypotension, and hypovigilance) in response to inescapable physical and psychological stressors (Keay and Bandler 2015). Our prior work has described VLPAG as a node of the somatomotor-sympathetic circuitry that likely contains direct projections to spinal cord (Kerman et al. 2006; Kerman 2008). Although small numbers of somatomotor-sympathetic projections are also observed in DLPAG and DMPAG, immunolabeling of these efferents is not observed until later survival times when viral infection reveals more upstream components of this circuitry (Kerman 2008).

In the present study, our PRV tract-tracing investigation revealed that WKY rats (compared to Sprague Dawley rats) display significantly fewer somatomotor efferents in several subdivisions of the PAG (dorsomedial, lateral, and ventrolateral), as well as a trend for a decrease in these efferents in DLPAG. In bLR rats (compared to bHR rats), significant reductions in immunolabeling of premotor efferents were limited to the DLPAG (although statistically trending decreases were also observed in LPAG and VLPAG). It is pertinent to note that interpretation of the findings in bLR rats is complicated by the overall modest levels of viral labeling in these animals. One possibility suggested by the data is that the tendency of these strains to display passive stress coping behaviors reflects impairments in the ability to mount an active coping strategy (as opposed to favoring a passive strategy) due to a reduction in functional connections sufficient to engage peripheral motor targets following psychogenic or physical stressors. In the context of existing behavioral assessments in rodent models, active stress coping strategies (e.g., increased swimming or climbing in the FST) by typically behaving rodents may be influenced by psychological (via the DLPAG) or physical/homeostatic (via the LPAG) challenges associated with the testing experience, or potentially a combination of these factors. By contrast, the relevance of reduced DMPAG or VLPAG premotor efferents in male WKY rats, as described in the present study, is less clear. The DMPAG is involved in mediating defensive responses to social threats (Faturi et al. 2014; Franklin et al. 2017), which do not appear to be studied in the WKY rat strain. (Decreased sociability under non-stressful conditions is already a reported feature in WKY rats (Nam et al. 2014).) The VLPAG, in addition to its role in mediating passive coping to inescapable stressors (Keay and Bandler 2015), is also implicated in rapid eye movement (REM) sleep regulation (Kroeger et al. 2019), food intake (Hao et al. 2019), and pain modulation (Sun et al. 2020). While disturbances relevant to each of these functional domains are reported in depressed patients, it is not possible to identify how altered premotor circuits from DMPAG or VLPAG may contribute to these specific perturbations in the WKY model based on the scope of our current work.

The findings of our PRV tract-tracing investigations in LC and PAG should be interpreted with consideration of several methodological limitations. Firstly, survival times following initial injections of PRV-152 varied between experiments, with bHR/bLR rats surviving 120 h post-injection and WKY/SD rats surviving 132 h post-injection. These survival times correspond to short and intermediate survival times in our group’s prior work (Kerman et al. 2006). Survival times of 120 h are sufficient to identify somatomotor-sympathetic efferents in PVN, LH, ventrolateral PAG, and ventromedial medulla. These efferents are likely to project directly to the spinal cord and collateralize to innervate somatic motoneurons and sympathetic preganglionic neurons simultaneously. At intermediate and later survival times, dual-labeled neurons appear in additional brain structures such as the DMH and different subdivisions of the PAG (Kerman 2008). While the neuroanatomical investigations described here do not intend to draw direct comparisons between the WKY and bLR strains, it should be noted that without examining neuroanatomical characteristics at both time points for each strain, it is not possible here to draw conclusions about whether a particular neuroanatomical area has direct or indirect projections to spinal cord (or to other nodes of the somatomotor-sympathetic circuitry) in the respective strains. Secondly, overall immunolabeling of somatomotor circuits in our experiments with bHR and bLR rats was considerably diminished relative to that of WKY and Sprague Dawley rats. Experiments in the bHR/bLR model were carried out later than those in the WKY/SD strains, and disparities in viral labeling are likely due to inevitable decreases in viral titer that occur naturally over extended periods in storage. Finally, the experiments in the present study were conducted only in male animals. It is important to acknowledge that behavioral and symptomatic presentations of depression and anxiety, as well as treatment efficacy for these conditions, vary between sexes in animal models and in humans (Marcus et al. 2005; Bigos et al. 2009; Dalla et al. 2010; LeGates et al. 2018). Work in animal models demonstrates that certain indices in various emotional behavioral assessments are more appropriate for one sex versus the other (e.g., latency to immobility in females versus immobility duration in males in Porsolt’s FST) and that female and male rodents engage differential stress coping mechanisms (Kokras et al. 2009; Kokras and Dalla 2017). Where or to what extent premotor circuit alterations are observed in male versus female rodents could play a significant role in modulating their disparate behavioral phenotypes. Furthermore, the observation that defensive coping strategies differ between sexes may be particularly salient with respect to our neuroanatomical findings in the PAG, given the known role of this structure in modulating active and passive stress coping strategies (Bandler et al. 2000; Keay and Bandler 2015).

## Conclusions

The LC and PAG are involved in coordinating behavioral and autonomic responses to stress. Deficits in premotor projections from these structures, particularly the dorsolateral and lateral PAG, have the potential to contribute to the passive coping strategies in response to stressful stimuli. The neuroanatomical data presented in this report expand our knowledge of how premotor efferents in central nervous system structures implicated in somatomotor-sympathetic integration are altered in two distinct rodent models for heritable differences in stress reactivity and emotional behavior. In particular, male subjects from the WKY and bLR rat lines display fewer polysynaptic projections to skeletal muscle from the LC and various subregions of the PAG. These data support the idea that while the overall behavioral inhibition profile in bLR and WKY rats is generated by a set of largely overlapping circuits, there remain unique features between these strains. More broadly, these results may enhance our understanding of how emotional motor circuits are dysregulated in individuals suffering from clinical depression or anxiety-related disorders.

## Data Availability

The data that support the findings of this study are available from the corresponding author upon reasonable request.
